# Evaluation of the efficacy of platelet rich fibrin (I-PRF) intra-articular injections in the management of internal derangements of temporomandibular joints – a controlled preliminary prospective clinical study

**DOI:** 10.1186/s12891-022-05421-7

**Published:** 2022-05-14

**Authors:** Mustafa Manafikhi, Jawdat Ataya, Omar Heshmeh

**Affiliations:** 1 Oral and Maxillofacial Department, Faculty of Dentistry, Damascus University, Damascus, Syria; 2 Medical Education Programme, Syrian Virtual University, Damascus, Syria; 3 Faculty of Dentistry, Damascus University, Damascus, Syria; 4 Oral and Maxillofacial Department, Faculty of Dentistry, Al-Sham Syrian Private University, Damascus, Syria

**Keywords:** Injectable platelet rich fibrin (I-PRF), Temporomandibular disorders, Temporomandibular joint, Intra-articular injections

## Abstract

**Background:**

The temporomandibular joint (TMJ) is one of the most complex joints in the body. Temporomandibular disorders (TMD) are among the most troublesome disorders for patients, as they can cause pain, affect oral functions and disturb dentists on the level of diagnosis and treatment. The most common symptoms of temporomandibular joint disorders are articulated sounds (such as clicking or TMJ crepitation), joint pain in comfort and function situations (jaw movements), pain or strain in masseter and jaw muscles and or restricted mandibular movements. One of the most modern biocompatible substances used to treat joint disorders, including the TMJ, is platelet-rich plasma (PRP) and injectable platelet-rich fibrin (I-PRF). This study aims to evaluate the efficacy of platelet-rich fibrin (I-PRF) intra-articular injections in managing internal derangements of temporomandibular.

**Methods:**

Twenty patients suffering from a unilateral click due to temporomandibular disorders were individually injected with 1 mL of (I-PRF) twice 1 week apart into the superior joint space of the TMJ with the internal disorder. Data were recorded and evaluated by the Helkimo index. This evaluation was conducted three times; 1 week after the first injection, 1 week after the second injection, and 6 months after the first injection.

**Results:**

The clicking disappeared entirely in 14 out of 20 patients after 1 week of getting the first injection, and in all patients after 1 week of getting the second injection, and returned to two of them after a six-month since the first injection.

**Conclusion:**

Preliminary results showed the efficacy of the Injectable Platelet-Rich Fibrin (I-PRF) in managing articular clicking in patients with internal disorders of the temporomandibular joint. Injectable Platelet Rich Fibrin has significant clinical efficacy in treating the articular clicking resulting from internal temporomandibular joint disorders.

## Background

The temporomandibular joint (TMJ) is one of the most complex joints in the body [1]. Temporomandibular disorders (TMD) are among the most troublesome disorders for patients, as they can cause pain, affect oral functions and disturb dentists on the level of diagnosis and treatment [2]. Several studies have discussed and developed several classifications of TMJ disorders [3, 4].

The most common symptoms of temporomandibular joint disorders are articulated sounds (such as clicking or TMJ crepitation), joint pain in comfort and function situations (jaw movements), pain or strain in masseter and jaw muscles and or restricted mandibular movements [5]. In this study, TMJ sounds are highlighted.

TMJ sounds are divided into clicking and TMJ crepitation that is resulted from the friction of bone surfaces and is often associated with the irreversible anterior displacement of TMJ [6]. Joint clicking can occur for several reasons [7]. Distinguishing among its types is highly important as each one has its clinical significance and a particular way to be addressed [7, 8]. The TMJ clicking is considered the most likely to be refractory to treatment [8]. Changes in anatomical shape, TMJ hypermobility, muscular asymmetry and articular disc displacement affect its types [7, 8].

TMDs often accompany stress, anxiety, depression, and psycho-emotional stress, so they are often present in college students during exams [9, 10] or even during the COVID-19 pandemic [11], according to several studies [12–16]. Symptoms of TMJ disorders were present in adolescents [17], and myofascial pain was the most prevalent type. TMD was significantly more common in girls [18].

One of the most important causes of TMJ disorders is the biopsychosocial situation [19, 20], which indicates economic, psychological stress, and psychological diseases such as depression [21]. Therefore, knowledge of patients exposed to TMD is useful in accelerating treatment and targeting them with awareness and preventive programs [7].

In previous studies, injections of steroids in the jaw joint have been frequently suggested [2, 22]. Nevertheless, they have shown that injecting such substances into a narrow space such as the TMJ leads to irritation and erosion of articular surfaces [23].

Therefore, there are several alternative therapies for TMD, such as low-intensity pulsed ultrasound (LIPUS) [24], Low-Level Laser Therapy (LLLT) [25], Biofeedback in Masticatory Muscle Activity Management [26], Transdermal Cannabidiol Application [27], Myorelaxant of bee venom topical skin [28], Electroacupuncture for Temporomandibular Disorders which confirmed by a systematic review [29] that it was more effective in combination with other interventions than when conducted alone.

One of the most modern biocompatible substances used to treat joint disorders, including the TMJ, is platelet-rich plasma (PRP) [23]. Platelet-rich plasma (PRP) has recently been a necessary treatment for joints. It is also a modern therapy with advantages exceeding corticosteroids in treating degenerative and necrotic TMD [23]. Platelet-rich plasma (PRP) has anti-inflammatory, antibiotic and analgesic properties [23]. It helps restore intra-articular acidic acid and stimulates cartilage cells to produce Glycosaminoglycans [23].

It also regulates the balance of angiogenesis within the joint [23]. Several evidence-based studies demonstrated that PRP induces cell proliferation and cartilage production through cartilage cells [30]. In addition, it increases the production of hyaluronic acid through synovial cells [23, 30].

According to several peer-reviewed scientific papers [31–33], the uses of PRP and PRF in dentistry are many, especially in the field of endodontics, periodontal treatments, oral surgery, and others, because of their role in the regeneration process and regenerative medicine.

A randomized controlled clinical study [31] conducted at Damascus University by Al-Kurdi et al. demonstrated the efficacy of I-PRF in reducing the resorption of fat following facial lipostructure. In several animal laboratory studies, experiments showed that platelet-rich plasma facilitates the repair of cartilaginous and osteochondral lesions [34, 35]. One- year of observation after intra-articular injections of the knee showed the clinical benefit of platelet-rich plasma in alleviating pain for patients with knee pain [34]. In addition, platelet-rich plasma treatment achieves high concentrations of a group of growth factors that accelerate healing, including the transforming growth factor β, which is responsible for cartilage formation in cartilage restoration [34]. Platelet-rich plasma has been used in regenerative dentistry as it provides high physiological concentrations of endogenous growth factors that can induce tissue regeneration [36]. However, there are still concerns about anticoagulants [37]. Results of a previous study [37] showed that growth factors in platelet-rich plasma were higher in an early stage, whereas injectable platelet-rich fibrin, used in this study, showed significantly high levels of growth factors such as IGF_1, EGF, PDGF_AA, PDGF_AB in the long run [37]. Ten days after the injection, both concentrates showed high biocompatibility [37]. Moreover, injectable platelet-rich fibrin showed high levels of mRNA of growth factors: TGF_B within 7 days, PDGF within 3 days and collagen on the third and seventh days compared with platelet-rich plasma [37].

The study showed that injectable platelet-rich fibrin (I-PRF) could release high concentrations of different growth factors, such as transforming growth factor β, compared to platelet-rich plasma [37]. Recently plasma derivatives have been used, including platelet-rich plasma [37]. They were initially injected into the knee joint and then into the temporomandibular joint, and good results were obtained [37].

This study aims to evaluate the efficacy of platelet-rich fibrin (I-PRF) intra-articular injections in managing internal derangements of temporomandibular. This study has no controlled group.

## Methods

A prospective clinical study was conducted between January 2020 and February 2021; the first registration was on 14/01/2020 in the Department of Oral and Maxillofacial Surgery outpatient clinics, Faculty of Dentistry, Damascus University. Besides, we got a registration number from the Australian New Zealand Clinical Trials Registry (ANZCTR) as Retrospectively registered, 08/03/2022 ACTRN12622000397718. After informing the patients about the purpose and nature of the research study verbally and in writing, ensuring their understanding and answering their questions, written consent was obtained from all participants.

The sample size was calculated according to the program (G Power 3.1.7 V.), considering that the significance level was 5%, the study power was 90%, the effect size was 1.39 mm, and the maximum standard deviation was 7.68 mm. The information was input and processed into the program, and the total sample size was 20 patients.

The inclusion criteria were that the patients should be between 16 and 60 years old, all patients in the sample should not have systemic diseases that may affect the healing of soft and bone tissue, patients should not have undergone any previous surgeries in the TMJ, and the patient should have unilateral clicking in the temporomandibular joint. Patients should not have syndromes or growth disorders, osteoarthritis of the TMJ (advanced stages), TMJ disk perforation, rheumatism, and both types of TMJ intra-articular adhesion, infectious arthritis, and pregnant women or patients with bilateral arthralgia.

**Fig. 1:**
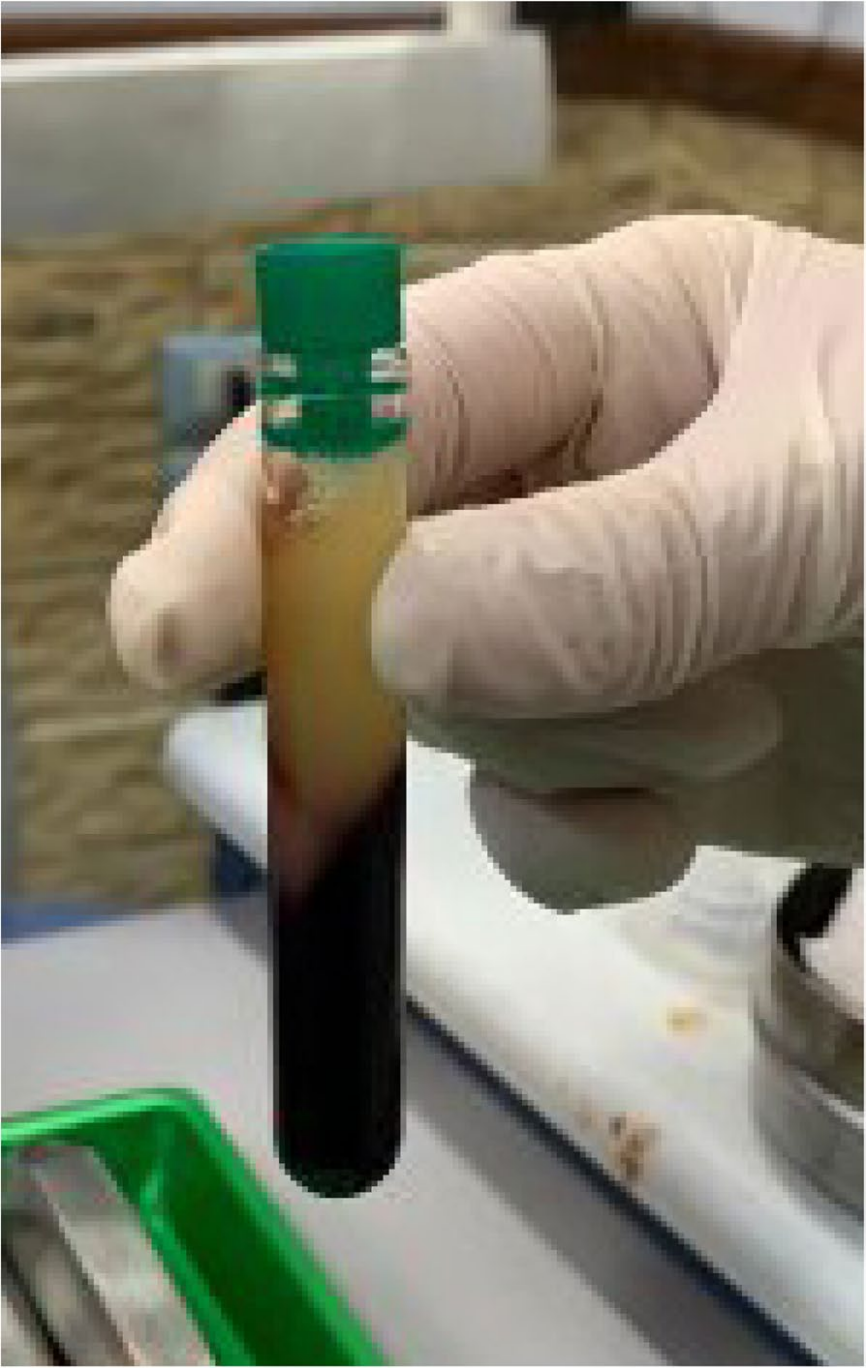
Injectable Platelet Rich Fibrin (I-PRF)

Patients were asked to tell their own medical and dental history during their study visit, which will spend 20 to 30 minutes. Observations were recorded in the patient’s medical examination forms, and the clicking was evaluated using the Helkimo index. These values ​​were recorded in a patient form before injecting the TMJ with I-PRF. The researcher conducted a clinical examination of the temporomandibular joint, and this study was based on a functional manual examination of the temporomandibular joint [8]. This simple and comprehensive method was used to diagnose the symptoms of the temporomandibular joint with special emphasis on negating and neutralizing the effects of muscles in developing symptoms. For injectable platelet-rich fibrin preparation, prepared by a device centrifuge (Hettich® EBA 20 centrifuge, Sigma-Aldrich), 20 mL peripheral blood was obtained from the ulnar vein and collected in sterile vacuum tubes used one time. These tubes were put in a centrifuge at 700 rpm for 3 minutes [37]. Thus, we got platelet concentrates in liquid form and not in the form of clots. This liquid is rich in white blood cells, platelets and growth factors and can increase healing properties [38]. There were no additives Figure 1. shows Injectable Platelet Rich Fibrin (I-PRF).

For the injection technique by a medical syringe, an oral surgeon disinfected the surgical site of ​​the TMJ with povidone. The entry point of the needle tip was determined by delimiting the anatomical features of the entry point, which was suggested by McCain [39].

We started with a line extending from the middle of the tragus to the lateral angle of the eye on the same side. The injection needle was entered about (10) mm from the middle of the pinna tragus and about (2) mm down the previously drawn line. An Insulin syringe was used and entered wholly (27 gauge in diameter, approximately 1 cm in length). The injection continued until a patient felt the pressure of the injection, and the plunger turned back as the finger was removed. The injection was stopped, and the needle was drawn immediately. There were no other forms of TMD treatment at the time of the study implementation.

The following injection protocol was used. Each patient has injected twice at 1 mL 1 week apart. One week after the first injection, patients were examined, the results were recorded, and the second injection was performed in the same joint and with the same method. Since the second injection, patients were followed for 6 months. The clicking was evaluated by the Helkimo scale [40].

Degrees of TMJ movement according to the Helkimo index and corresponding values for each degree which is as follows: 0 scores for no joint sounds or deviation < 2 mm during mouth opening or closing, one score for articulated noises or deviation > 2 mm during mouth opening or closing and five score for obstruction or dislocation of the joint.

The data entry and analysis were performed using Statistical Package for Social Sciences software package (SPSS Inc., Chicago, IL, USA) version 23.

Several statistical tests were carried out. Initially, Friedman’s analysis was done, and the *p*-value was equal to 0.00. Therefore, the Wilcoxon analysis was done, and the *p*-value was according to Table 2.

## Results

The sample consisted of 20 patients suffering from a click in unilateral TMDs. The sample consisted of 7 (35%) males and 13 (65%) females. The average age was 27.4, with 11.3 for Stander Deviation.

**Fig. 2:**
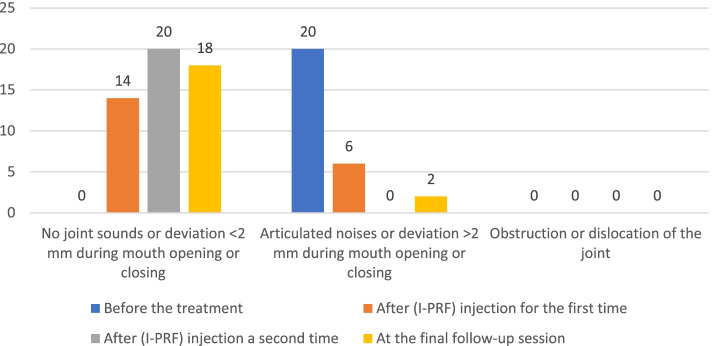
Shows the results of the TMJ clicking according to the Helkimo index

The results showed that the clicking disappeared in 16 (80%) patients after the first injection, and all 20 (100%) patients had no clicking after the second injection. Nevertheless, the clicking was returned to two patients after a 6-month follow-up. Therefore, the results of the TMJ clicking according to the Helkimo index are in Table 1 and Fig. 2.

**Table 1:** Shows the results of the TMJ clicking according to the Helkimo index

	Before the treatment N (%)	After (I-PRF) injection for the first time N (%)	After (I-PRF) injection a second time N (%)	At the final follow-up session N (%)
No joint sounds or deviation < 2 mm during mouth opening or closing	0 (0%)	14 (70)	20 (100%)	18 (90%)
Articulated noises or deviation > 2 mm during mouth opening or closing	20 (100%)	6 (30%)	0 (0%)	2 (10%)
Obstruction or dislocation of the joint	0 (0%)	0 (0%)	0 (0%)	0 (0%)
The sum	20 (100%)	20 (100%)	20 (100%)	20 (100%)

Friedman’s analysis was performed to study the relationship between TMJ clicking and duration, the *P*-value was 0.00, which is less than 0.05, and therefore there is a significant effect, while the Wilcoxon analysis was conducted to compare the different periods, which appear in Table 2.

**Table 2:** The results of Wilcoxon test

Comparison of the two periods:	*P*-value	Statistical significant
After (I-PRF) injection for the first time - before treatment	0.000	**Yes**
After (I-PRF) injection a second time - before treatment	0.000	**Yes**
At the final follow-up session - before processing	0.000	**Yes**
After fibrin injection for the second time - after (I-PRF) injection for the first time	0.014	**Yes**
At the final follow-up session - after (I-PRF) injection for the first time	0.102	No
At the final follow-up session - after fibrin injection for the second time	0.157	No

## Discussion

In recent years, there has been a growing tendency to use injectable platelet-rich fibrin and platelet-rich plasma for treating various internal disorders of the TMJ due to its therapeutic benefits. This study aims to introduce a modern method for treating the symptoms of internal temporomandibular joint disorders. It assessed the benefits of this method and the efficacy of injecting the TMJ with Injectable Platelet-Rich Fibrin (I-PRF).

In this research, Injectable Platelet Rich Fibrin (I-PRF) was used. This substance does not entail adding external factors such as anticoagulants, unlike platelet-rich plasma. It also allows the release of growth factors over a more extended time than platelet-rich plasma [37].

The presence and absence of the TMJ clicking were assessed by the Helkimo index within a score range that determines the movement of the temporomandibular joint. The study showed the absence of TMJ clicking between pre-treatment and post-injection periods and at the final follow-up 6 months after the second injection.

At a 95% confidence level, there were statistically significant differences between the pre-treatment period and the first injection. In addition, significant differences were found in the period between the first and the second injection. There were no statistically significant differences between the second injection and the final follow-up, and that means there was an improvement in the absence of clicking between the pre-treatment and the post-first injection periods. An improvement in the absence of clicking was also noticed between the second and the first injection. The improvement between the final follow-up and the second injection did not change.

These results were consistent with the clinical study done by Savina Gupta et al. (2018) [41], who compared injecting platelet-rich plasma and hydrocortisone with local anaesthetic within the temporomandibular joint to manage temporomandibular joint disorders.

Our findings contrast with Hegab et al. at Al-Azhar University in Egypt, who conducted a clinical study on the effect of platelet-rich plasma (PRP) on TMJ arthritis [23]. They concluded that the clicking completely disappeared in all research subjects during a follow-up period of 1 year [23]. In our study, clicking reappeared in two patients after a follow-up period of 6 months. The reason behind that may be using different injection protocols. In Hegab’s study, the injection was performed three times at a week interval. However, two injections were performed in our research at a week interval. Thus, the third injection may positively affect the absence of clicking during a long follow-up period.

Our findings were similar to the clinical study by Hanci et al. 2015 who evaluated the efficacy of injecting platelet-rich plasma in TMD management and compared it with TMJ arthrocentesis [42]. The study included 20 patients (15 females, five males) with a reversible disk displacement in 32 TMJs. Platelet-Rich Plasma proved to be highly influential in alleviating pain, improving mouth opening, and treating articular sounds compared to TMJ arthrocentesis.

A study found that the combination of hyaluronic acid with PRP helps improve patients with TMDs if we compare it with hyaluronic acid alone [43].

The limitation of the study is the lack of a control group.

## Conclusions

Injectable Platelet Rich Fibrin (I-PRF) injection into the TMJ leads to the absence of the articular clicking as one of the internal temporomandibular disorders (TMD) during a 6-month follow-up period. Injectable Platelet Rich Fibrin has substantial clinical efficacy in treating the articular clicking resulting from internal temporomandibular joint disorders.

## Data Availability

All the necessary data are presented herewith. However if needed, raw data on excel format can be available on request from the corresponding author.

## Abbreviations

TMJ: The temporomandibular joint
TMD: The temporomandibular Disorders
I-PRF: Injectable Platelet Rich Fibrin
PRP: Platelet-Rich Plasma

## References

[1] Alomar X, Medrano J, Cabratosa J, Clavero JA, Lorente M, Serra I (2007). Anatomy of the temporomandibular joint. Seminars in Ultrasound, CT and MRI.

[2] Freund BJ, Schwartz M (2002). Use of botulinum toxin in chronic whiplash-associated disorder. Clin J Pain.

[3] Peck CC, Goulet JP, Lobbezoo F, Schiffman EL, Alstergren P, Anderson GC (2014). Expanding the taxonomy of the diagnostic criteria for temporomandibular disorders. J Oral Rehabil.

[4] Schiffman E, Ohrbach R, Truelove E, Look J, Anderson G, Goulet J-P (2014). Diagnostic criteria for temporomandibular disorders (DC/TMD) for clinical and research applications: recommendations of the international RDC/TMD consortium network* and orofacial pain special interest group†. J Oral Facial Pain Headache.

[5] Dimitroulis G (2018). Management of temporomandibular joint disorders: a surgeon’s perspective. Aust Dent J.

[6] Derwich M, Lassmann L, Machut K, Zoltowska A, Pawlowska E. General characteristics, biomedical and dental application, and usage of chitosan in the treatment of temporomandibular joint disorders: a narrative review. Pharmaceutics. 2022;14(2):305. 10.3390/pharmaceutics14020305 PMC8880239 35214037

[7] Bair E, Rathnayaka N, Diatchenko L, Greenspan JD (2019). Clinical predictors of persistent temporomandibular disorder in people with first-onset temporomandibular disorder. J Am Dent Assoc.

[8] Gauer R, Semidey MJ (2015). Diagnosis and treatment of temporomandibular disorders. Am Fam Physician.

[9] Lung J, Bell L, Heslop M, Cuming S, Ariyawardana A. Prevalence of temporomandibular disorders among a cohort of university undergraduates in Australia. J Investig Clin Dent. 2018;9(August 2017):1–5. 10.1111/jicd.12341 29604182

[10] Wu J, Huang Z, Chen Y, Chen Y, Pan Z, Gu Y. Temporomandibular disorders among medical students in China : prevalence, biological and psychological risk factors. BMC Oral Health. 2021;21:1–8. 10.1186/s12903-021-01916-2 PMC8549286 34702237

[11] Medeiros RA De, Vieira DL, Vivianne E, Santos RW, Tabata LF. Prevalence of symptoms of temporomandibular disorders, oral behaviors, anxiety, and depression in Dentistry students during the period of social isolation due to COVID-19 Abstract. 2020;28:1–8. 10.1590/1678-7757-2020-0445 PMC7714260 33263648

[12] Emodi-Perlman A, Eli I, Smardz J, Uziel N, Wieckiewicz G, Gilon E, et al. Temporomandibular disorders and bruxism outbreak as a possible factor of orofacial pain worsening during the COVID-19 pandemic-concomitant research in two countries. J Clin Med. 2020;9(10):3250. 10.3390/jcm9103250 PMC7601612 33053640

[13] Wieckiewicz M, Grychowska N, Nahajowski M, Hnitecka S, Kempiak K, Charemska K, et al. Prevalence and overlaps of headaches and pain-related temporomandibular disorders among the polish urban population. J Oral Facial Pain Headache. 2019;34:1–9. 10.11607/ofph.2386 31465030

[14] Valesan LF, Da-cas CD, Réus JC, Cristina A, Denardin S, Garanhani RR, et al. Prevalence of temporomandibular joint disorders : a systematic review and meta-analysis. Clin Oral Investig. 2021;25:441–53. 10.1007/s00784-020-03710-w 33409693

[15] Lins N, Arnaud M, Goretti M, Lima DS, Guimar S, Gomes F (2019). Prevalence of TMD and level of chronic pain in a group of Brazilian adolescents.

[16] Wieckiewicz M, Grychowska N, Wojciechowski K, Pelc A, Augustyniak M, Sleboda A (2014). Prevalence and correlation between TMD based on RDC/TMD diagnoses, oral parafunctions and psychoemotional stress in Polish university students. Biomed Res Int.

[17] Graue AM, Jokstad A, Assmus J, Skeie MS. Prevalence among adolescents in Bergen , Western Norway , of temporomandibular disorders according to the DC / TMD criteria and examination protocol. Acta Odontol Scand. 2016;6357(June):449–55. 10.1080/00016357.2016.1191086 27251463

[18] Mara F, Bertoli DP, Bruzamolin CD, Pizzatto E, Losso M, De SJF (2018). Prevalence of diagnosed temporomandibular disorders : a cross-sectional study in Brazilian adolescents.

[19] Sharma S, Breckons M, Brönnimann Lambelet B, Chung J, List T, Lobbezoo F (2020). Challenges in the clinical implementation of a biopsychosocial model for assessment and management of orofacial pain. J Oral Rehabil.

[20] Ohrbach R, Dworkin SF (2016). The evolution of TMD diagnosis: past, present, future. J Dent Res.

[21] Dıraçoǧlu D, Yıldırım NK, Saral I, Özkan M, Karan A, Özkan S (2016). Temporomandibular dysfunction and risk factors for anxiety and depression. J Back Musculoskelet Rehabil.

[22] Stoustrup P, Kristensen KD, Verna C, Küseler A, Pedersen TK, Herlin T (2013). Intra-articular steroid injection for temporomandibular joint arthritis in juvenile idiopathic arthritis: a systematic review on efficacy and safety. Semin Arthritis Rheum.

[23] Hegab AF, Ali HE, Elmasry M, Khallaf MG (2015). Platelet-rich plasma injection as an effective treatment for temporomandibular joint osteoarthritis. J oral Maxillofac Surg Off J Am Assoc Oral Maxillofac Surg.

[24] Namera MO, Mahmoud G, Abdulhadi A, Burhan A (2020). Effects of low-intensity pulsed ultrasound (LIPUS) applied on the temporomandibular joint (TMJ) region on the functional treatment of class II malocclusion: a randomized controlled trial. Dent Med Probl.

[25] Madani A, Ahrari F, Fallahrastegar A, Daghestani N (2020). A randomized clinical trial comparing the efficacy of low-level laser therapy (LLLT) and laser acupuncture therapy (LAT) in patients with temporomandibular disorders. Lasers Med Sci.

[26] Florjanski W, Malysa A, Orzeszek S, Smardz J, Olchowy A, Paradowska-Stolarz A, et al. Evaluation of biofeedback usefulness in masticatory muscle activity management-a systematic review. J Clin Med. 2019;8(6):766. 10.3390/jcm8060766 PMC6616888 31151198

[27] Nitecka-Buchta A, Nowak-Wachol A, Wachol K, Walczyńska-Dragon K, Olczyk P, Batoryna O, et al. Myorelaxant effect of transdermal Cannabidiol application in patients with TMD: a randomized, double-blind trial. J Clin Med. 2019;8(11):1886. 10.3390/jcm8111886 PMC6912397 31698733

[28] Nitecka-buchta A, Buchta P, Tabeńska-Bosakowska E, Walczyńska-Dragoń K, Baron S (2014). Myorelaxant Effect of Bee Venom Topical Skin Application in Patients with RDC / TMD Ia and RDC / TMD Ib : a randomized , double blinded study.

[29] Sung S, Kim D, Park M, Hwang S, Yoon Y, Park J (2021). Electroacupuncture for temporomandibular disorders : a systematic review of randomized controlled trials.

[30] Park H-B, Yang J-H, Chung K-H (2011). Characterization of the cytokine profile of platelet rich plasma (PRP) and PRP-induced cell proliferation and migration: upregulation of matrix metalloproteinase-1 and-9 in HaCaT cells. Korean J Hematol.

[31] Alkerdi K, Alsabek L, Alkhouli M, Al-Nerabieah Z, Jaafo H (2022). Evaluation of the effect of injectable platelet-rich fibrin (I-PRF) in reducing the resorption of fat graft during facial lipostructure: a randomized clinical trial. Dent Med Probl.

[32] Bhattacharya HS, Gummaluri SS, Astekar M, Gummaluri RK (2020). Novel method of determining the periodontal regenerative capacity of T-PRF and L-PRF: an immunohistochemical study. Dent Med Probl.

[33] Pietruszka P, Chruścicka I, Duś-Ilnicka I, Paradowska-Stolarz A. PRP and PRF-subgroups and divisions when used in dentistry. J Pers Med. 2021;11(10):944. 10.3390/jpm11100944 PMC8540475 34683085

[34] Kavadar G, Demircioglu DT, Celik MY, Emre TY (2015). Effectiveness of platelet-rich plasma in the treatment of moderate knee osteoarthritis: a randomized prospective study. J Phys Ther Sci.

[35] Graziani F, Ivanovski S, Cei S, Ducci F, Tonetti M, Gabriele M (2006). The in vitro effect of different PRP concentrations on osteoblasts and fibroblasts. Clin Oral Implants Res.

[36] Albanese A, Licata ME, Polizzi B, Campisi G (2013). Platelet-rich plasma (PRP) in dental and oral surgery: from the wound healing to bone regeneration. Immun Ageing.

[37] Miron RJ, Fujioka-Kobayashi M, Hernandez M, Kandalam U, Zhang Y, Ghanaati S (2017). Injectable platelet rich fibrin (i-PRF): opportunities in regenerative dentistry?. Clin Oral Investig.

[38] Fujioka-Kobayashi M, Katagiri H, Kono M, Schaller B, Zhang Y, Sculean A, et al. Improved growth factor delivery and cellular activity using concentrated platelet-rich fibrin (C-PRF) when compared with traditional injectable (i-PRF) protocols. Clin Oral Investig. 2020;24(12):4373–83. 10.1007/s00784-020-03303-7 32382929

[39] McCain JP, Hossameldin RH (2011). Advanced arthroscopy of the temporomandibular joint. Atlas Oral Maxillofac Surg Clin North Am.

[40] Helkimo M (1974). Studies on function and dysfunction of the masticatory system. IV. Age and sex distribution of symptoms of dysfunction of the masticatory system in Lapps in the north of Finland. Acta Odontol Scand.

[41] Gupta S, Sharma A, Purohit J, Goyal R, Malviya Y, Jain S (2018). Comparison between intra-articular platelet-rich plasma injection versus hydrocortisone with local anesthetic injections in temporomandibular disorders: a double-blind study. Natl J Maxillofac Surg.

[42] Hancı M, Karamese M, Tosun Z, Aktan TM, Duman S, Savaci N (2015). Intra-articular platelet-rich plasma injection for the treatment of temporomandibular disorders and a comparison with arthrocentesis. J Cranio Maxillo Facial Surg Off Publ Eur Assoc Cranio Maxillo Facial Surg.

[43] Harba AN, Harfoush M (2021). Evaluation of the participation of hyaluronic acid with platelet-rich plasma in the treatment of temporomandibular joint disorders. Dent Med Probl.

